# Brain Docosahexaenoic Acid [DHA] Incorporation and Blood Flow Are Increased in Chronic Alcoholics: A Positron Emission Tomography Study Corrected for Cerebral Atrophy

**DOI:** 10.1371/journal.pone.0075333

**Published:** 2013-10-02

**Authors:** John C. Umhau, Weiyin Zhou, Shantalaxmi Thada, James Demar, Nahed Hussein, Abesh K. Bhattacharjee, Kaizong Ma, Sharon Majchrzak-Hong, Peter Herscovitch, Norman Salem, Abigail Urish, Joseph R. Hibbeln, Stephen C. Cunnane, Stanley I. Rapoport, Jussi Hirvonen

**Affiliations:** 1 Laboratory of Clinical and Translational Studies, National Institute on Alcohol Abuse and Alcoholism, National Institutes of Health, Bethesda, Maryland, United States of America; 2 PET Department, National Institutes of Health, Bethesda, Maryland, United States of America; 3 Brain Physiology and Metabolism Section, Laboratory of Neurosciences, National Institute on Aging, National Institutes of Health, Bethesda, Maryland, United States of America; 4 Laboratory of Membrane Biochemistry and Biophysics, National Institute on Alcohol Abuse and Alcoholism, National Institutes of Health, Bethesda, Maryland, United States of America; 5 Department of Medicine, University of Sherbrooke, Sherbrook, Quebec, Canada; 6 Department of Radiology and Turku PET Centre, University of Turku, Turku, Finland; Centre for Addiction and Mental Health, Canada

## Abstract

**Objective:**

Chronic alcohol dependence has been associated with disturbed behavior, cerebral atrophy and a low plasma concentration of docosahexaenoic acid (DHA, 22∶6n-3), particularly if liver disease is present. In animal models, excessive alcohol consumption is reported to reduce brain DHA concentration, suggesting disturbed brain DHA metabolism. We hypothesized that brain DHA metabolism also is abnormal in chronic alcoholics.

**Methods:**

We compared 15 non-smoking chronic alcoholics, studied within 7 days of their last drink, with 22 non-smoking healthy controls. Using published neuroimaging methods with positron emission tomography (PET), we measured regional coefficients (K*) and rates (*J_in_*) of DHA incorporation from plasma into the brain of each group using [1-^11^C]DHA, and regional cerebral blood flow (rCBF) using [^15^O]water. Data were partial volume error corrected for brain atrophy. Plasma unesterified DHA concentration also was quantified.

**Results:**

Mean K* for DHA was significantly and widely elevated by 10–20%, and rCBF was elevated by 7%–34%, in alcoholics compared with controls. Unesterified plasma DHA did not differ significantly between groups nor did whole brain *J_in_*, the product of K* and unesterified plasma DHA concentration.

**Discussion:**

Significantly higher values of K* for DHA in alcoholics indicate increased brain avidity for DHA, thus a brain DHA metabolic deficit vis-à-vis plasma DHA availability. Higher rCBF in alcoholics suggests increased energy consumption. These changes may reflect a hypermetabolic state related to early alcohol withdrawal, or a general brain metabolic change in chronic alcoholics.

## Introduction

Chronic alcohol dependence has been associated with disturbed behavior, cerebral atrophy and a low plasma concentration of the polyunsaturated fatty acid (PUFA) docosahexaenoic acid (DHA, 22∶6n-3), particularly if liver disease is present [1], [2]. Furthermore, reduced dietary n-3 PUFA was reported to promote binge alcohol induced neurodegeneration [3], and a low plasma DHA concentration was associated with relapse vulnerability in substance abusers [4]. In animal models, excessive chronic alcohol intake was reported to lower DHA concentration in blood and nervous system; the effect was worsened by a low n-3 PUFA diet [5], [6].

DHA at high concentrations are found in the stereospecifically numbered (*sn*-2) of brain membrane phospholipids, particularly ethanolamine glycerophospholipid and phosphatidylinositol. DHA in *sn*-2 position can be hydrolyzed by a phospholipase A_2_ (PLA_2_), particularly by Ca^2+^-independent iPLA_2_, during neurotransmission and other processes [7], [8], . Once released, some DHA is metabolized to bioactive products such as neuroprotectin D1 and resolvins, which have anti-inflammatory roles [11]. DHA and its metabolites also modulate gene expression, neurotransmission, enzyme activity, membrane channels, receptor activation, inflammation and other cellular processes [12], [13], [14].

DHA cannot be synthesized *de novo* by the body, nor elongated significantly in brain from its circulating nutritionally-essential precursor, alpha-linolenic acid (α-LNA, 18∶3n-3) [15]. Thus the brain’s DHA comes directly from the diet or from ingested a-LNA that has been elongated and desaturated in the liver [16]. Liver disease may interfere with this conversion and result in less circulating DHA available for brain incorporation [1], [17].

Most of the DHA that is released from membrane phospholipids by a PLA_2_ is rapidly reincorporated into available lysophospholipid [18]. It is possible to quantitatively image the rate of loss, as it equals the net rate of DHA incorporation from plasma, *J_in_*. *J_in_* can be measured in vivo using positron emission tomography (PET) following intravenous infusion of positron-labeled [1-^11^C]DHA [19], as the product of unesterified plasma DHA concentration and a DHA incorporation coefficient, K* [18]. K* measures the brain’s affinity or avidity for circulating DHA for the replacement of DHA loss by metabolism [15], [20]. K* represents several different steps, including DHA diffusion from plasma to brain and into brain cells, intracellular DHA acylation to DHA-CoA by an acyl-CoA synthetase with the consumption of 2 ATPs, and DHA transfer from DHA-CoA to membrane lysophospholipid by an acyltransferase [21], [22], [23]. K* is independent of changes in regional cerebral blood flow (rCBF) (flow can be doubled using CO_2_ inhalation without changing K*), and is determined by exchange kinetics with circulating albumin [14], [24], [25]. On the other hand, rCBF is a marker of brain functional activity, is coupled to regional brain glucose metabolism [26], and can be quantitatively imaged using PET with [^15^O]water [27]. rCBF is reported to be reduced in alcoholics [28].

The animal and humans studies noted above suggest that brain DHA metabolism is altered in chronic alcoholics. We thought it of interest to directly test this suggestion by quantifying regional brain DHA incorporation using PET and [1-^11^C]DHA, in recently sober alcoholics compared with healthy controls, using our established PET method and model [18], [19]. Animal studies show that following the intravenous infusion of labeled DHA, 90% of tracer taken up is esterified in phospholipids within a few minutes, whereas only 10% is β-oxidized. Thus, the label enters the recycling step of DHA turnover [8], [18].

We also thought it important to measure rCBF with [^15^O]water, as a marker of overall energy metabolism and functional activity [26], [29]. To do this, we combined an injection of [^15^O]water followed after its radioactivity largely disappeared (^15^O half life is 2 min) by infusion of [1-^11^C]DHA (half-life 20.3 min) in the same PET session. Because of reported brain atrophy in chronic alcoholics, we corrected DHA incorporation and rCBF values for the partial volume effect (PVE) of atrophy [30]. We hypothesized that the alcoholics would exhibit altered DHA metabolism and rCBF on PET when compared to controls. Results from a subset of the control sample are presented in a preliminary report [19].

## Materials and Methods

### Human Subjects

The National Institute on Alcohol Abuse and Alcoholism (NIAAA) institutional review board specifically approved this study (Protocol No. 04-AA-0058), as did the National Institutes of Health (NIH) Radiation Safety Committee. Written informed consent was obtained from participants, who were compensated for their participation. We studied 15 alcoholics and 22 healthy controls between 19 and 65 years of age, who were recruited from the Bethesda, Maryland area. Controls were non-smokers and reported no medication, drug or alcohol use for at least 2 weeks prior to the PET scan. Non-smoking alcoholics were selected from the population of alcoholics admitted for acute alcohol detoxification to the NIAAA inpatient ward at the NIH Clinical Center in Bethesda, Maryland. We studied non-smokers to reduce the possible confounding effects and increased variability associated with smoking [31], [32]. The alcoholics were scanned within 7 days of their last drink of alcohol, but were not exhibiting signs of acute withdrawal. We studied only alcoholics who did not require pharmacological treatment for alcohol withdrawal, and who were not taking psychoactive medication. All subjects had negative toxicological urine screens for drugs of abuse, and no history of intravenous drug abuse. All subjects underwent an extensive history and physical examination with laboratory tests to ensure that they were free of significant medical problems and had no history of neurological or psychiatric disorders other than alcoholism. A structured research questionnaire was employed to obtain information on lifetime alcohol consumption [33].

We performed PET after first injecting [^15^O]water to image rCBF, [1-^11^C]DHA to image regional brain DHA incorporation coefficients K*, as described in detail elsewhere [19]. Scans on a subject were acquired at approximately 11 a.m. following 24 h on a standardized low DHA diet and an overnight fast. We collected blood three times during the scan to quantify plasma unesterified fatty acid concentrations. Fifteen minutes following the injection of a bolus of [^15^O]water, 1118±24 MBq (30.2±0.7 mCi) [1-^11^C]DHA was infused intravenously for 3 minutes at a constant rate (Harvard Infusion Pump, South Natick, MA). Because of the high specific activity the [1-^11^C]DHA (see above), less than 0.06 µmol unlabeled DHA was infused into a subject; thus there was no significant pharmacological effect. Serial dynamic 3-D scans were acquired during the hour following the start of infusion. Arterial blood samples (2–5 ml) were obtained at fixed times to determine radioactivity in whole blood and plasma. In addition, a subset of samples was used to measure blood [^11^C]CO_2_, and the parent fraction of [1-^11^C]DHA.

### Determination of Plasma [1-^11^C]DHA Input Function (Plasma Curve)

To rapidly assay plasma [1-^11^C]DHA during a PET scan, we used a solid phase extraction procedure to separate unesterified [1-^11^C]DHA from remaining plasma radioactivity. From plasma samples collected at 0, 3, 7, 10, 15, 20, 40 and 60 minutes post-infusion of [1-^11^C]DHA, total lipids were extracted into chloroform: methanol (1∶1) [34]. The original method was modified to allow for a rapid single-step extraction due to ^11^C decay. Briefly, 0.3 ml of plasma was placed into glass centrifuge screw-cup tubes containing a mixture of 1 ml methanol, 1 ml of chloroform, and 0.6 ml of water. The tubes were purged with nitrogen, sealed, vigorously, vortexed for 30 seconds, and centrifuged at 4,000 rpm for 5 minutes. The bottom layer containing the total lipid extract was collected by aspiration. Using this single pass extraction, recovery of available total lipids from the plasma sample was found to be in the range of 50–60%, with the remainder still found in the aqueous phase. Recovery of lipids from plasma for this extraction procedure was determined in a separate experiment using ^14^C- and ^3^H-labeled individual lipid probes. Solid phase extraction analysis was carried out on 500 mg aminopropylsilane (NH_2_) cartridges (BAKERBOND spe™; JT Baker, Phillipsburg, NJ) according to a method adapted from Agren [35]. The total lipid extracts were evaporated to complete dryness under a stream of nitrogen gas in a heating block maintained at 45°C. The lipid extracts were dissolved in 0.25 ml of hexane-MTBE-acetic acid (100∶3:0.3) loading solvent. A 0.1 ml aliquot of this solution was reserved for counting and 0.1 ml was applied onto solid phase extraction cartridges for separation into lipid classes. The separations were performed on a vacuum manifold under 30–40 mm Hg vacuum. Four to ten samples were separated simultaneously. Prior to separation, the solid phase extraction cartridges were pre-conditioned with 10 ml of hexane. Combined cholesterol ester and triglyceride fractions were eluted with 10 ml of hexane-chloroform (2∶1), non-esterified fatty acids with 10 ml of chloroform-methanol-acetic acid (100∶2:2) and phospholipids with 8 ml of isopropanol-3N methanolic HCl (4∶1).

All lipid fractions were collected and counted with a calibrated gamma counter. This procedure also was applied to a reference blood sample taken before injection, to which approximately 185 MBq (5 µCi) of [1-^11^C]DHA was added. Of the original plasma total lipid extract loaded on the solid phase extraction column, greater than 98% of the counts were recovered in the non-esterified fatty acid fraction (second elution step), and very few counts, if any, were associated with the plasma triglycerides, cholesteryl ester, and phospholipids. The final continuous function for the [1-^11^C]DHA fraction was determined as the product of two fitted curves, one for the time-varying recovery and one for the non-esterified fatty acid fraction determined by the ratio of the second fraction to the sum of the fractions. The continuous functions were chosen based on those described previously [30].

To verify the identity of the tracer determined from the solid phase extraction procedure, we conducted a separate procedure on a subset of samples. HPLC was performed on the unesterified plasma fatty acid fraction from the second elution step to determine percent radioactivity due to [1-^11^C]DHA. Measurements were made on 5-ml plasma samples at 10 minutes post-infusion of [1-^11^C]DHA, as well as on plasma spiked with [1-^11^C]DHA. The fractions were dried under nitrogen gas, redissolved in methanol, and separated by high performance liquid chromatography (System Gold ® model 126, Beckman; Fullerton, CA) using a 25 cm×4.6 mm i.d., C18 reverse phase column (Luna (I) ™, Phenomenex; Torrence, CA). Elution (2 ml/min) of unesterified fatty acids was by a linear gradient of acetonitrile/15 mM H_3_PO_4_ in water, initiated and held at 80∶20 (v/v) for 1 minute, increased to 96∶4 (v/v) in 10 minute, held at 96∶4 (v/v) for 10 minutes, and returned to 80∶20 (v/v) in 1 minute [36]. Elution was monitored at 192 nm with an ultraviolet/visible light detector (Model 151, Gilson; Middleton, WI). Radioactivity profiles were obtained with an on-line flow scintillation counter (β-Ram, model 2B, IN/US Systems, Tampa, FL) using a 2∶1 ratio of scintillation cocktail (IN-FLOW™ 2∶1, IN/US Systems) to monitor high performance liquid chromatography column outflow. The radioactive signal was monitored using a Laura Lite 3 computer program (version 3.2, IN/US Systems, Lab Logic Systems Ltd). Following the manufacturer’s instructions, the counting window was set at 80–1000 keV to capture both gamma and beta particle emissions from [^11^C] decay events. Peaks were identified against retention times of unlabeled standards of unesterified fatty acids. Recovery of unesterified fatty acids through the high performance liquid chromatography column was 95–98%.

For co-registration of PET scans to brain anatomy, we obtained a magnetic resonance (MR) image of the brain with a 1.5 Tesla Horizon scanner (General Electric, Milwaukee, WI). This produced T1-weighted volumetric spoiled gradient magnetic resonance (MR) images for superimposition onto the PET images, which were used to register both rCBF images from the [^15^O]water scans and [1-^11^C]DHA parametric images. Appropriate registration of [1-^11^C]DHA/[^15^O]water images on to the MR images was visually verified. Because of the limited spatial resolution of a PET scan, underestimation of radioactivity can occur in high-activity gray matter regions. To provide the most accurate measure of activity in specific regions of gray matter, we corrected for this partial volume effect (PVE). This correction is particularly important when studying disorders associated with cerebral atrophy, such as alcoholism and Alzheimer disease [37], [38]. It provides a better measure of actual tissue metabolism or flow free of effects of cerebrospinal fluid, and corrects for loss of the radioactive signal to adjacent tissue and for spill-in of signal from adjacent tissue [37], [38], [39].

### Regions of Interest (ROI)

Regions of interest (ROI) were drawn manually on individual MR images on six continuous axial MR slices [19]. Non-PVE corrected values of K* and rCBF, as well as PVE-corrected values, were obtained for gray matter regions from PET images by limiting averaging to voxels identified as gray matter by the segmentation procedure. Global gray matter K* and rCBF values were determined by averaging all voxels in the gray matter mask. Values for K* and rCBF for white matter were obtained from the PET images by limiting the averaging to voxels identified as 99% pure white matter from the smoothed white matter mask [19].

### Statistics

Data were analyzed using SPSS Statistics 17.0 for Windows (Release 17.0.0, copyright SPSS Inc., 1993–2007). K* and *J_in_* means were compared between groups using a mixed model two-way analysis of variance (ANOVA), with group status (alcoholics *vs.* controls) as between-subject factor and brain region (gray matter regions) as within-subject factor. The groups did not differ in terms of gender (χ^2^ = 1.3, p = 0.247), race (χ^2^ = 2.3, p = 0.684) or body mass index (BMI), (t = −0.6, p = 0.547), but the patients tended to be older (44±14 years) than the controls (36±14 years), although not significantly (p = 0.089). Nevertheless, the analyses were repeated with age as a covariate. Significant main effects were followed up by regional T tests. P-values smaller than 0.05 were considered statistically significant. Means ± SD are given.

Corrections for multiple comparisons were not made because we are searching for overall patterns of changes in the brain, and because this was an exploratory study. Corrections are not recommended for such multipoint imaging studies, as they markedly increase type II errors [40], [41].

## Results

Table 1 presents characteristics of the two groups. For the alcoholics, the age of onset of heavy drinking at a mean of 26.9 years occurred about 17 years before PET scanning was performed.

**Table 1:** Descriptive variables.

Variables	Alcoholics	Controls
N	15	22
Gender Distribution	11 males	12 males
	4 females	10 females
Race	8 Caucasian	13 Caucasian
	5 African American	7 African American
	2 Other	2 Other
Age (y)	44±14	36±14
Weight (kg)	82±12	81±18
Body Mass Index (kg/m^2^)	27.3±3.7	26.4±4.7
Days sober before PET scan	4.8±1.6	N/A
Age of onset of heavydrinking (y)	26.9±10.6	N/A
Lifetime Alcohol drinking (kg)	383±343	3.5±4.8*

* p<0.001, data available for 21 controls.

### DHA Incorporation Coefficient K*

**Figure 1:**
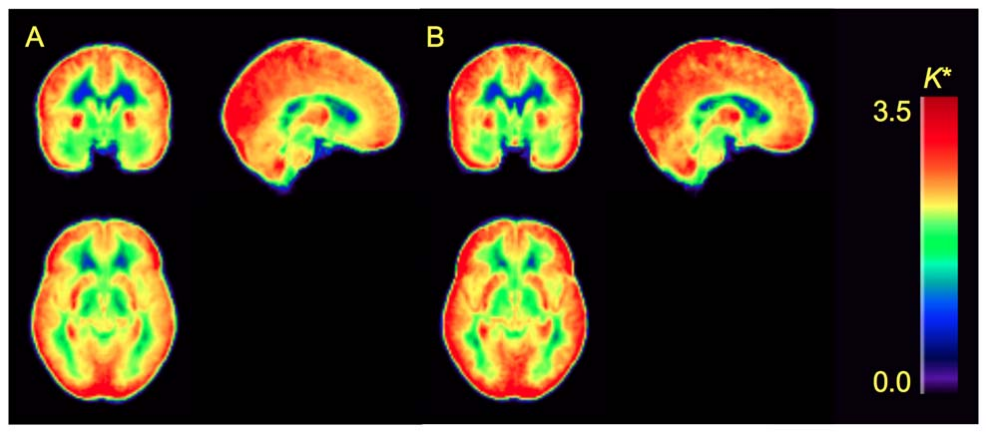
Parametric images of voxel-wise K* values for [1-^11^C]DHA. Images are group averages for controls (A; N = 22) and alcoholics (B; N = 15). Units are µL min^−1^ml^−1^ and are shown on color scale.

**Figure 2:**
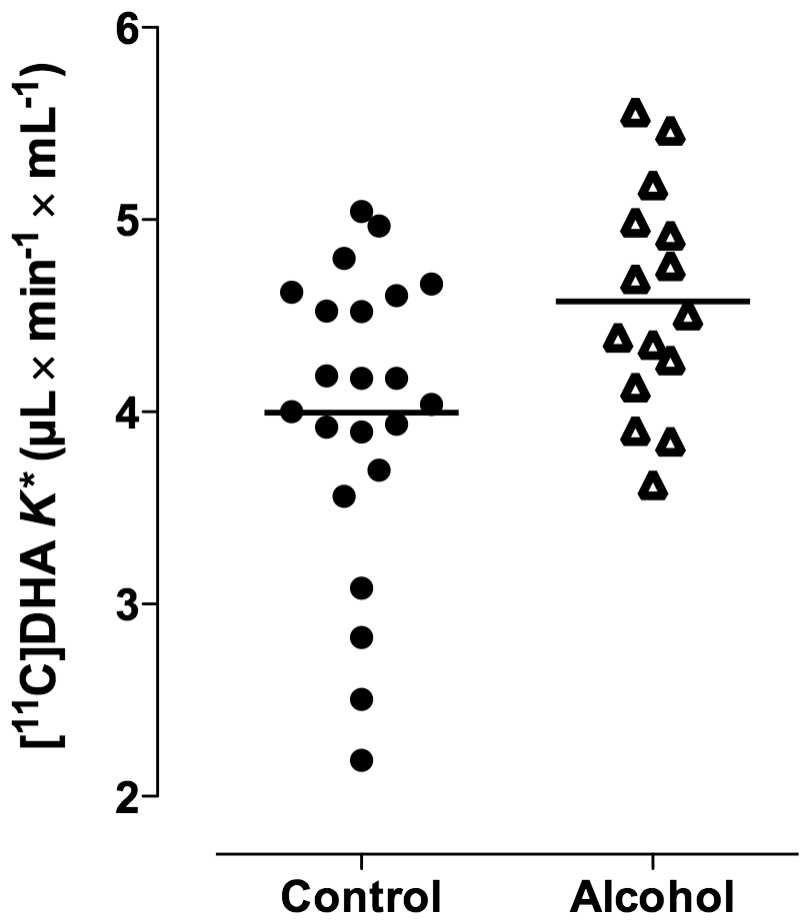
Significantly higher global gray matter K* for [1-^11^C]DHA in alcoholics vs. controls. (t-test p = 0.025; rmANOVA main effect of group, p = 0.041).

Mean and regional values of K* for DHA were significantly higher in alcoholics than in controls (main effect of group: F = 5.35, p = 0.027) (Figures 1 and 2, Table 2). Since the group×region interaction was insignificant (F = 1.7, p = 0.125), the higher K* was similar across brain regions (Table 2). Including age as a covariate did not change the results (main effect of group: F = 4.45, p = 0.042). K* was 10–20% higher in alcoholics among cortical regions, 20% higher in the thalamus, 17% higher in the cerebellar hemispheres, and 12% higher in the striatum. Among alcoholics, average K* in gray matter regions was not correlated with days of sobriety prior to the PET study (R^2^ = 0.06, p = 0.363). Mean K* in gray matter regions also did not correlate with body mass index (R^2^ = 0.01, p = 0.730, and R^2^ = 0.001, p = 0.867 respectively). Similarly, gender had no effect on average K* in gray matter regions in patients (t = 0.80, p = 0.439) or controls (t = 0.39, p = 0.699). K* did not differ according to race in alcoholics (F = 0.30, p = 0.824) or controls (F = 0.28, p = 0.839).

**Table 2:** Comparisons of regional values of *K* *and rCBF between alcoholics and controls.

Region	Controls		Alcoholics		Controls		Alcoholics	
	*K* * (µL min^−1^ml^−1^)				rCBF (ml min^−1^100 g^−1^)			
	Mean	±SD	Mean	±SD	Mean	±SD	Mean	±SD
Orbitofrontal	4.8	±0.9	5.6	±1.7	71	±17	75	±12
Prefrontal	4.0	±0.8	4.7*	±1.0	69	±14	81*	±15
Premotor	4.1	±0.6	4.7*	±0.8	83	±22	98*	±14
Anterior cingulate	3.0	±0.6	3.2	±0.5	68	±17	82*	±13
Inferior temporal	3.7	±0.7	4.1	±0.6	66	±14	74	±10
Middle temporal	3.8	±0.7	4.3*	±0.6	64	±15	74*	±8
Superior temporal	3.5	±0.8	3.7	±0.6	54	±13	62*	±9
Medial temporal	2.3	±0.5	2.5	±0.4	44	±7	51**	±9
Sensorimotor	4.2	±0.9	4.9*	±0.9	77	±15	103***	±14
Inferior parietal	4.1	±0.8	4.9**	±0.8	66	±13	81**	±15
Superior parietal	4.5	±0.9	5.1*	±0.8	64	±18	73	±18
Medial parietal	3.9	±0.8	4.3	±0.7	75	±15	89*	±17
Posterior cingulate	3.4	±0.8	3.8	±0.6	71	±15	85**	±16
Occipital association	4.3	±0.9	4.7	±0.7	68	±15	76	±13
Calcarine	4.6	±01.0	5.3*	±1.1	88	±21	110**	±24
Thalamus	4.1	±0.9	4.9**	±0.9	85	±21	109**	±27
Striatum	3.8	±0.9	4.2	±0.8	80	±18	94*	±19
Cerebellar hemisphere	3.5	±0.8	4.1*	±0.6	62	±11	78***	±12
Cerebellar vermis	3.2	±0.8	3.5	±0.6	56	±13	73***	±13
99% pure white matter	1.2	±0.3	1.3	±0.2	19	±4	21	±4
Global gray matter	4.0	±0.8	4.6*	±0.6	69**	±12	83**	±11
Average of 19 gray regions	3.8	±0.7	4.4*	±0.6	69**	±13	83**	±11

* p<0.05;

** p<0.01;

*** p<0.001, differs from control mean.

### DHA Incorporation Rate J_in_

Daily incorporation rate *J_in_* of DHA into whole brain was calculated using the global (both white and gray matter) value for K* before PVE correction, and mean plasma concentrations of unesterified DHA. These concentrations were 1.83±0.78 nmol/mL in patients and 2.05±1.3 nmol/mL, and did not differ significantly (p = 0.547). In alcoholics, global *J_in_* equaled 5.3±2.3 µmol/day/g in alcoholics and 5.8±4.1 µmol/day/g brain for controls, and also did not differ significantly. Taking whole brain volume as determined by MR, 1196 ml for the alcoholics and 1242 ml for the controls, these incorporation rates are equivalent to DHA incorporation of 2.1±0.9 mg/day for alcoholics and 2.4±1.6 mg/day for controls for the whole brain (p = 0.520). These means are not significantly different, as might be expected since plasma DHA concentration tended to be reduced in patients compared with controls, whereas K* was increased.

### Blood Flow

Mean global CBF was significantly higher in alcoholics than in controls (main effect of group: F = 11.1, p = 0.002). The magnitude of this difference varied significantly between regions (group×region interaction: F = 2.94, p = 0.007). The main effect of group persisted even after including age as a covariate (F = 11.8, p = 0.002). The incremental differences in rCBF between alcoholics and controls ranged from 7% and 34% in cortical regions, and were 28% in the thalamus, 26% in the cerebellar hemispheres, and 18% in the striatum (Table 2).

Among alcoholics, average CBF in gray matter regions was not correlated with days of sobriety prior to the PET study (R^2^ = 0.11, p = 0.232). Body mass index correlated negatively with global CBF among alcoholics (R^2^ = 0.38, p = 0.015), but not among controls (R^2^ = 0.07, p = 0.228). Gender had no significant effect on average CBF in gray matter regions in alcoholics (t = −0.44, p = 0.667) or in controls (t = −1.7, p = 0.111). CBF did not differ according to race in alcoholics (F = 0.44, p = 0.728) or in controls (F = 0.31, p = 0.816). When considering the whole study sample, PVE correction raised values of global CBF by approximately 56%. When the original global CBF, uncorrected for the PVE, was compared between the two groups, the alcoholics had only 6% higher global mean CBF than the controls; this difference was not statistically significant (main effect of group: F = 3.32, p = 0.077).

Mean regional values for K*/rCBF were not significantly different between groups (main effect of group: F = 0.17, p = 0.682; group×region interaction: F = 0.49, p = 0.727). K* did not correlate with rCBF in any of the 20 regions identified in Table 1 among the alcoholics, but significant positive correlations were found in three of the 20 ROIs, sensorimotor cortex, thalamus, and striatum, in the controls.

## Discussion

We used PET with intravenously infused [1-^11^C]DHA and an irreversible uptake model to determine regional brain incorporation coefficients K* and to estimate rates *J_in_* for DHA in 15 recently sober, non-smoking alcoholics compared with 22 healthy non-smoking controls. We also injected [^15^O]water to measure rCBF prior to the K* measurements in the same PET session. Major findings were significantly higher mean values of K* for DHA in the alcoholics compared with controls, by 10–20%, and significantly higher values of rCBF (by 7%–34%). Mean unesterified plasma DHA concentration and mean *J_in_* for DHA did not differ between groups. rCBF was not correlated with K* in alcoholics in any of 20 ROIs, but was correlated significantly in only 3 of the 20 regions in controls. BMI correlated negatively with CBF in alcoholics but not controls.

The mean control values for K* and *J_in_* in this study do not differ significantly from previously published means in the subgroup of 14 subjects [19]. For interspecies comparison, the DHA incorporation coefficient (K*) and rate (*J_in_*) in unanesthetized male rats on a dietary adequate n-3 PUFA diet are K 10.8 µl.min^−1^.g^−1^ and Jin 21.6 nmol.day^−1^g^−1^, respectively [42], compared with 2–4.8 µl.min^−1^.ml^−1^ and 5.8±4.1 µmol/day/g brain our PET controls. This agrees with evidence that the overall body metabolic rate is about 2.5 fold higher in rats than in humans [29], and is consistent with ATP-dependent DHA incorporation into brain phospholipid via acyl-CoA synthetase [43].

We interpret the significantly higher values of K* in the alcoholics as likely representing increased avidity of the brain for circulating unesterified DHA. As noted in the Introduction, K* represents combined diffusional and enzymatic steps involved in DHA incorporation from plasma into brain phospholipid, replacing the DHA that has been metabolically lost [18], [20], [21]. K* was increased when plasma DHA concentration was reduced by severe (three generations) or moderate (12 weeks in one generation) dietary n-3 PUFA restriction in rats [12], [44]. Although we did not detect a lower plasma DHA concentration in the alcoholics at the scan time, with their typically poor dietary habits and frequent liver dysfunction, chronic alcoholics often have reduced plasma concentrations [45]. In the alcoholics, the elevated K* for DHA also might have arisen from disturbed or inefficient brain lipid metabolism, increasing the need for circulating DHA [46], [47], [48], [49]. To the extent that an increased K* represents disturbed brain DHA lipid metabolism directly or secondarily to reduced circulating DHA, it might be considered as a biomarker of such disturbance.

Regional values of K* for DHA were not correlated significantly with rCBF in the alcoholics, consistent with independence of brain PUFA uptake on rCBF [14], [24]. In the controls, positive correlations in only three of twenty regions could have been due to chance, or to the fact that DHA reacylation is energy consuming, and that rCBF reflects overall brain energy consumption [22], [26], [49].

Our finding significantly higher PVE-corrected CBF in the alcoholics who were in early abstinence differs from reports of reduced rCBF in chronic alcoholics as measured with PET [28], ^133^Xe clearance [50], [51], or single photon emission computed tomography (SPECT) [52], [53], [54]. PET also demonstrated reduced brain glucose metabolism in alcoholics studied 6 to 22 days after discontinuation of drinking [55].

There are possible several reasons for the discrepancy. One is that cigarette smoking can reduce rCBF [31], [32]. None of our alcoholics or controls was a smoker, but the other studies examining rCBF or glucose metabolism in alcoholics did not also exclude nonsmokers. When 80% of alcoholics in a study sample smoke [52], it is difficult to separate effects of smoking from the effects of chronic alcoholism on CBF.

Another possible reason for the difference between ours and others’ CBF findings is that the Xe-133 clearance and SPECT techniques used in some studies do not have as high resolution and do not provide absolute CBF values as does PET [56], [57]. Further, the CBF PET study included unmedicated hypertensive patients [28], but hypertension by itself reduces CBF [58]. In that study, patients were scanned while performing a cognitive task, there was a biphasic relation between CBF and self-reported drinking, and there was no control group. The PET study of glucose metabolism [55] reported cortical atrophy and ventricular enlargement in the alcoholics compared with controls, but did not correct for the PVE effect. Without the PVE correction, atrophy within a brain volume (voxel) reduces the estimated net radioactivity derived from the parenchyma [59], and atrophy correction is all the more necessary [37], [38], [60]. Although whole brain volume on MR in the present study was not significantly different between alcoholics and controls, regional atrophy likely occurred, as it is common in chronic alcoholics [55], [61].

Although our results remain to be confirmed, there are possible mechanisms for higher rCBF and regional K* for DHA in the alcoholics. Alcoholism can cause mitochondrial dysfunction [62], perhaps leading to a compensatory increase in rCBF. Also, since the alcoholics were studied within 7 days after their last drink, some may have experienced a hypermetabolic brain state, with a hyperactive EEG and increased risk for seizures [63], even though they did not exhibit signs of acute withdrawal (see Methods). This issue might be tested in the future studying patients at different times during abstinence when also obtaining EEG records. Disturbed brain metabolism is consistent with the significant negative correlation between body mass index and CBF in the alcoholics but not controls. A recent study showed a similar correlation between lower brain concentrations of N-acetylaspartate, choline containing compounds, creatine and phosphocreatine, markers of energy metabolism, and higher body mass, in alcoholics [64]. To the extent that an increased K* represents disturbed brain lipid metabolism, the PET measure of it might be considered as a biomarker of such disturbance.

In summary, our study provides baseline reference values for brain DHA incorporation and metabolism in nonsmoking healthy humans and alcoholics. It suggests that brain DHA incorporation and blood flow are elevated shortly after alcohol withdrawal in chronic alcoholics, and that PVE correction provides relevant data. Future studies using PET could determine if and when, in the course of recovery from alcoholism, brain DHA metabolism and CBF return to normal. Such studies may have important ramifications for the clinical care of recovering alcoholics.
